# DDANet: A deep dilated attention network for intracerebral haemorrhage segmentation

**DOI:** 10.1049/syb2.12103

**Published:** 2024-11-24

**Authors:** Haiyan Liu, Yu Zeng, Hao Li, Fuxin Wang, Jianjun Chang, Huaping Guo, Jian Zhang

**Affiliations:** ^1^ Department of Neurology Xinyang Central Hospital Xinyang China; ^2^ School of Medicine Xinyang Normal University Xinyang China; ^3^ School of Computer and Information Techonology Xinyang Normal University Xinyang China

**Keywords:** bioinformatics, image segmentation, learning (artificial intelligence), medical image processing, patient diagnosis

## Abstract

Intracranial haemorrhage (ICH) is an urgent and potentially fatal medical condition caused by brain blood vessel rupture, leading to blood accumulation in the brain tissue. Due to the pressure and damage it causes to brain tissue, ICH results in severe neurological impairment or even death. Recently, deep neural networks have been widely applied to enhance the speed and precision of ICH detection yet they are still challenged by small or subtle hemorrhages. The authors introduce DDANet, a novel haematoma segmentation model for brain CT images. Specifically, a dilated convolution pooling block is introduced in the intermediate layers of the encoder to enhance feature extraction capabilities of middle layers. Additionally, the authors incorporate a self‐attention mechanism to capture global semantic information of high‐level features to guide the extraction and processing of low‐level features, thereby enhancing the model's understanding of the overall structure while maintaining details. DDANet also integrates residual networks, channel attention, and spatial attention mechanisms for joint optimisation, effectively mitigating the severe class imbalance problem and aiding the training process. Experiments show that DDANet outperforms current methods, achieving the Dice coefficient, Jaccard index, sensitivity, accuracy, and a specificity of 0.712, 0.601, 0.73, 0.997, and 0.998, respectively. The code is available at https://github.com/hpguo1982/DDANet.

## Graphical abstract

This study presents DDANet, an innovative deep learning model specifically designed for segmenting intracerebral haemorrhage in brain CT images. The model incorporates dilated convolution pooling, self‐attention mechanisms, residual networks, and channel–spatial attention to enhance feature extraction and tackle class imbalance. Experiments show that DDANet surpasses current methods with a Dice coefficient of 0.712 and a Jaccard index of 0.601.

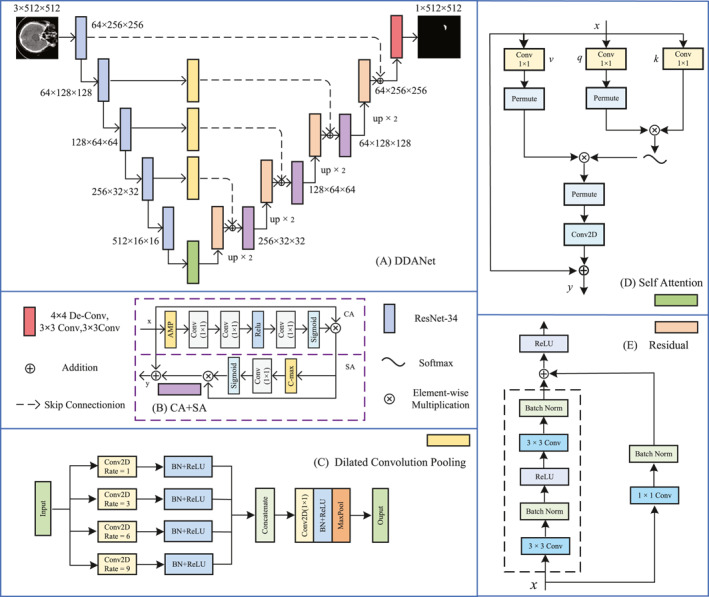

## 1 INTRODUCTION

Intracranial haemorrhage (ICH) is a life‐threatening brain disorder and one of the leading causes of stroke [1, 2]. ICH is extremely lethal, especially within 1 month of onset, with a mortality rate of up to 40% [3, 4]. Even if patients survive, there are many complications that lead to the development of severe disability. Data show that only about 20% of patients are able to return to independent living within 6 months [5]. In the early stages of cerebral haemorrhage, rapid and accurate diagnosis is critical for the survival and recovery of patients [6]. If physicians are able to quickly recognise the haemorrhage and accurately determine its location and extent, they can carry out effective interventions within the critical time window for treatment. This not only significantly improves the chances of survival but also reduces the risk of long‐term complications faced by patients [7]. Therefore, early diagnosis and precise localisation of the haemorrhagic area are an integral part of ICH treatment and are of great significance in improving the survival rate and quality of life of patients. In the diagnosis of cerebral haemorrhage, CT scanning is the technique of choice for emergency evaluation due to its rapidity, affordability, and high availability [8]. Although CT provides clear images of the brain and assists in identifying haemorrhagic areas, accurate segmentation of haemorrhagic areas usually requires manual work by experienced radiologists. This process is time‐consuming and highly dependent on the technical skills of the physicians [4]. Therefore, it is crucial to develop reliable automated segmentation techniques that not only improve diagnostic efficiency and precision but also minimise human error, allowing physicians to concentrate on more complex cases.

In recent years, with the rapid development of deep learning, particularly the application of deep convolutional neural networks (DCNNs) in medical image segmentation, significant progress has been made [9, 10, 11, 12, 13, 14, 15, 16, 17, 18]. These methods learn high‐level features automatically from training data, avoiding the labour‐intensive process of manually designing features. Many studies have focus on different types of deep learning models to tackle various challenges in medical image segmentation and these studies can be roughly grouped into the following categories:

(1) U‐Net and its variants: Models based on U‐Net and its variants dominate the field of medical image segmentation. U‐Net [11], as a representative of the encoder–decoder architecture, achieves great success in medical image segmentation. Its variants, such as U‐Net++ [19] and U‐Net with attention mechanisms [20], further improve segmentation accuracy by incorporating dense skip connections and attention mechanisms. These improvements, particularly in feature extraction and information flow, provide better performance for more complex medical image segmentation tasks.
(2) Residual and dilated convolution models: Models incorporating residual connections and dilated convolutions play a crucial role in enhancing segmentation performance. For example, BRU‐net proposed by Stefanos et al. [21] combines residual connections and dilated convolutions, significantly improving the segmentation accuracy of retinal Optical Coherence Tomography images. MA et al. [22] proposed a residual mixed convolution strategy for the task of haematoma segmentation in brain CT images, effectively addressing the degradation problem in small lesion areas. Similarly, InfiNet by Kumar et al. [23] and X‐Net by Qi et al. [24], which combine residual mechanisms and depthwise separable convolutions, achieve promising results in brain image segmentation tasks.
(3) Multi‐module and attention mechanism models: Models that introduce multi‐module structures and attention mechanisms demonstrate strong performance in multi‐class medical image segmentation. CE‐Net, proposed by Gu et al. [25], combines various module structures to capture multiscale information and global semantics, resulting in outstanding performance in various medical image segmentation tasks. The multiscale CNN algorithm proposed by Dutta et al. [26] further improves the recognition of key regions by introducing attention mechanisms, achieving higher accuracy in multi‐class brain segmentation tasks.

In previous research, although methods such as DCNNs have made significant progress in medical image segmentation, important challenges remain in practical clinical applications, particularly in haemorrhage detection in the brain. First, the shape, size, and location of haemorrhagic regions often exhibit considerable variation, which can cause existing models to perform poorly under large deformations [27]. Second, smaller lesions, such as minor haemorrhages, are often overlooked during segmentation, leading to reduced sensitivity in detecting these subtle abnormalities [28]. Finally, the contrast between haemorrhage regions and surrounding normal tissue is often low, making the segmentation results susceptible to interference from blurred boundaries. This is especially problematic in low‐contrast images, where the robustness of the model may be compromised [29]. These limitations collectively impact the performance of existing models in brain haemorrhage segmentation tasks, particularly when handling complex morphological variations, small lesions, and low‐contrast regions. Addressing these issues is crucial not only for enhancing model accuracy but also for promoting the widespread clinical application of medical image segmentation technologies.

To address the above issues, based on U‐Net, this paper proposes a novel *D*eep *D*ilated *A*ttention *Net*work called DDANet for tiny ICH segmentation. DDANet is an enhanced U‐Net framework, consisting of feature extraction and feature aggregation. For the feature extraction stage, we propose deeply expanded convolution pooling (DCP) modules to improve the depth and breadth of middle‐level features, aimed at enhancing the richness of mid‐level features. DCP allows the network to capture a wider range of contextual information while maintaining sensitivity to details by introducing the notion of dilation rate in convolutional operations. This mechanism effectively expands the receptive field, allowing the model to better understand complex structures and patterns in images, especially when dealing with medical images with high variability. In addition, we utilise the self‐attention module (SAM) to mine and preserve shallow semantic features, a strategy that effectively prevents the loss of semantic information of small‐scale objects during CNN processing. SAM, as an advanced mechanism, empowers DDANet to capture the global context beyond the restricted local sensory field of traditional convolutional layers. The introduction of self‐attention not only enriches the model's perception of local features but also significantly improves its ability to integrate global semantic information. For the feature aggregation stage, we reasonably restructure the residual architecture, channel attention and spatial attention mechanisms to achieve rapid and effective feature integration. The residual architecture [30] is to mitigate the vanishing gradient problem and enable the training of deeper networks, whereas the attention mechanism [31], on the other hand, including channel attention and spatial attention, can enhance the model's ability to recognise key features. This aggregation also effectively mitigates the severe class imbalance problem and aids the training process.

In summary, the main contributions of this study are as follows:

- We propose a novel end‐to‐end U‐Net called DDANet for the tiny ICH segmentation task. We design DCP and SAM to extract features with more rich spatial and semantic information. In particular, DCP enhances the depth and richness of mid‐level features, whereas SAM captures global semantic information of high‐level features, thereby enhancing the model's understanding of the overall structure.
- We reasonably refine the residual structure, channel attention and spatial attention mechanisms for feature aggregation, thereby enhancing the decoder features and accelerate the convergence of our DDANet. The residual structure is to mitigate the vanishing gradient problem and enable the training of deeper networks, whereas the attention mechanism enables the network to focus on key regions in the image.
- Rich and extensive experiments show that the Dice coefficient and Jaccard index of our DDANet are 0.712 and 0.601, respectively, both exceeding the industry average standards, indicating excellent precision and edge recognition in the ICH segmentation task.

The remainder of the paper is organised as follows: Section 2 introduces the dataset used for experiments, Section 3 describes the proposed DDANet, Section 4 presents the experimental results, Section 5 interprets the results of our study, and finally, the work is summarised in Section 6.

## 2 DATASETS

### 2.1 Dataset description

**FIGURE 1:**
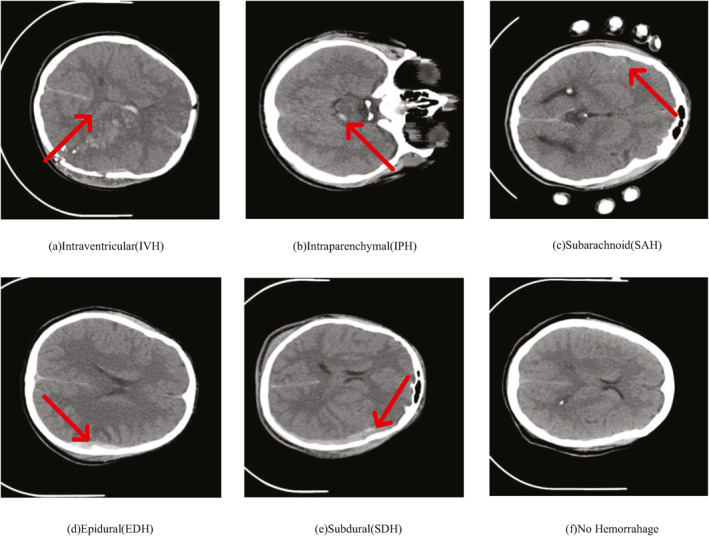
Images with the different types of ICH: (a) intraventricular (IVH); (b) intraparenchymal (IPH); (c) subarachnoid (SAH); (d) epidural (EDH); (e) subdural (SDH) and (f) no haemorrhage.

The ICH dataset used in this study was originally made publicly available in Ref. [17] with the data sourced from real clinical cases at the Al Hilla Teaching Hospital in Iraq. As shown in Figure 1, the uniqueness of this dataset lies in its coverage of various types of ICHs, including intraventricular haemorrhage (Figure 1a), intraparenchymal haemorrhage (Figure 1b), subarachnoid haemorrhage (Figure 1c), epidural haemorrhage (Figure 1d), subdural haemorrhage (Figure 1e) and no haemorrahage (Figure 1f). This dataset consists of CT scan samples from 82 patients, of which 36 samples were diagnosed with different types of haemorrhages, while the remaining 46 samples were normal cases without haemorrhage. The CT scanning equipment used was Siemens/SOMATOM Definition AS, ensuring that the images from each scan are of high quality and consistency. On average, each CT scan contains 30 slices, with a slice thickness of 5.0 mm, and a total of 1080 images of size 512×512 pixels were collected. Out of the 1080 images, 318 were identified with haemorrhagic spots and utilised to assess the model's performance. This dataset is proprietary but has been publicly released for specific research purposes, available to relevant researchers. The diversity and high quality of this dataset make it an ideal choice for ICH segmentation tasks.

### 2.2 Data pre‐processing

In order to evaluate the effectiveness of our proposed method in the task of cerebral haemorrhage (ICH) segmentation, we divide the dataset using five‐fold cross‐validation, which is an accurate technique for model evaluation. The dataset is evenly into five parts, reserving one part at a time as a test set for verifying the model's performance while the other four parts are combined for training the model. With five iterations, each rotating a different test set, we are able to ensure that every data point is involved in the test, resulting in a comprehensive and balanced model performance evaluation. Therefore, for each fold, the ratio of training and testing sets is 80%–20%. This approach not only improves the reliability of the evaluation results, but also helps us gain a deeper understanding of the model's performance under various data distributions, providing strong data support for model optimisation and selection.

In terms of data annotation, the ICH region on each CT slice was finely labelled by two experienced radiologists who jointly confirmed the ground truth labels. Since the original dataset has only 318 training images, the limited amount of data causes the deep learning model to be prone to overfitting. To solve this problem, we apply a series of data enhancement techniques to both images and labels, including random rotation, flipping, scaling, contrast transformation and noise interference. These methods help enrich the training samples and improve the generalisation ability of the model. This dual validation process ensure the precision and reliability of the labelling and provide high‐quality labelled data for model training. Training and testing on such a comprehensive and challenging dataset effectively assesses the model's capability to detect various types of cerebral haemorrhages and its adaptability to diverse patient profiles and scanning conditions.

## 3 METHODS

### 3.1 Overall architecture

**FIGURE 2:**
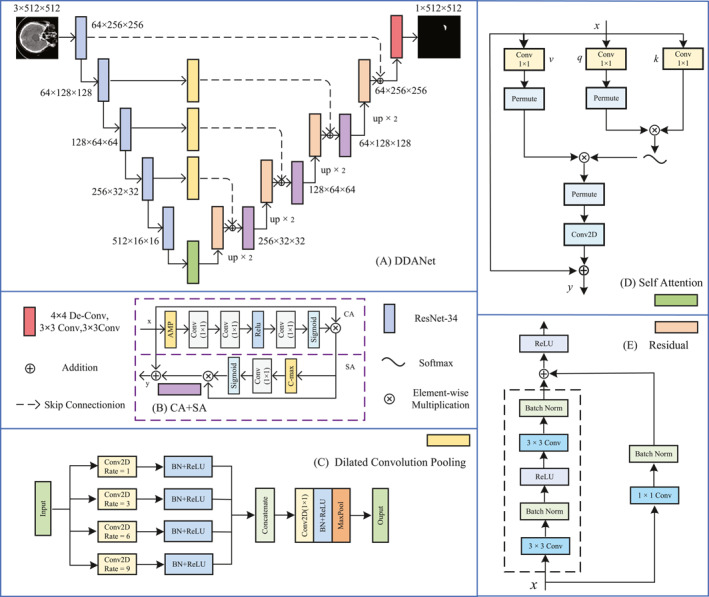
The architecture of the proposed DDANet.

Figure 2a shows the structure of our DDANet for the ICH segmentation task. In order to improve the model's generalisation and multiscale feature processing ability in the brain haemorrhage segmentation task, the brain haemorrhage images are first fed into the encoding network for processing. We adopt a modified U‐Net structure to improve the segmentation performance, in which the pre‐trained ResNet‐34[30] is used as the encoder network. Before information fusion, a proposed DCP strategy is employed to capture multiscale contextual information of the target. Except for the first level of the encoder, the DCP blocks are placed at each level of the encoder to enhance the receptive field of the network. Subsequently, SAM is introduced to capture global semantic information of high‐level features to guide the extraction and processing of low‐level features, thereby enhancing the model's understanding of the overall structure while maintaining details. Finally, using residual, spatial, and channel attention mechanisms during the decoding phase, the resolution of the feature maps is gradually restored, resulting in the final brain haemorrhage segmentation outcomes. More details of the DCP block, SAM, residual module, and channel and spatial attention mechanism are presented in Sections 3.2, 3.3, 3.4 and 3.5.

### 3.2 Dilated convolution pooling block

Considering the continuous stacking of downsampling in the encoding stage may lead to severe loss of important details, making it difficult to reconstruct information from small objects. Therefore, reducing the loss of critical information from small objects has become an urgent issue that needs to be resolved.

To mitigate the impact of the aforementioned issue, we propose the DCP block to further capture the multiscale contextual features of ICHs of different sizes, as shown in Figure 2c. Dilated Convolution Pooling cleverly utilises parallel 3×3 convolutional layers, applying different dilation rates to achieve multiscale processing of the input feature maps. This design not only expands the receptive field of the convolutional kernels but also allows the network to capture features at various levels by gradually increasing the dilation rate, thus generating richer and more detailed feature maps at each layer. After each convolutional layer, the combination of batch normalisation and ReLU activation function further optimises the training process, accelerates the convergence speed, and enhances the model's responsiveness to different features. By merging the outputs of the ReLU activation functions, we obtain a feature map that integrates multiscale information, providing a more comprehensive feature representation for the subsequent 1×1 convolutional layer. The use of the 1×1 convolutional layer effectively reduces the number of feature channels while maintaining the expressive power of the features, which is crucial for lowering the computational complexity and reducing the parameter scale. Subsequent batch normalisation and ReLU activation further enhance the non‐linear representation ability of the features. Finally, through dimensionality reduction by the max pooling layer, the model reduces computational burden while retaining key spatial information, providing a more compact and efficient feature representation for subsequent network layers. This carefully designed structure enables the DCP block to excel in feature extraction and dimensionality reduction, providing a solid foundation for the construction and optimisation of deep networks.

### 3.3 Self‐attention module

The self‐attention mechanism enhances the model's capture of global information and improves performance [32]. Here, we use self‐attention to explore semantic information cues of high‐level features to locate and guide low‐level information.

Figure 2d shows the details of our SAM. The main steps of SAM include generating the query, key, and value tensors through convolutions, as follows:

(1) $$q={\left(Reshape\left(Conv_{1x1}\left(x\right)\right)\right)}^{T}$$

(2) $$k=Reshape\left(Conv_{1x1}\left(x\right)\right)$$

(3) $$v=Reshape\left(Conv_{1x1}\left(x\right)\right)$$

where T is the matrix transpose operation. Then we obtain the attention weights using matrix multiplication and softmax computation. The output feature maps, weighted by these attention weights, are further convolved and summed with the original input to form the final output, as follows:

(4) $$y=X+Conv\left(Reshape\left(V⊗Softmax\left(q⊗k\right)\right)\right)$$

### 3.4 Residual block

Residual blocks are a key structure in deep learning networks, and are especially widely used in deep residual networks. As the network depth increases, simply stacking convolutional neural network (CNN) layers may hinder the training process and lead to the problem of exploding or vanishing gradients during backpropagation. To tackle this challenge, residual blocks introduce a shortcut connection alongside the network layers, as shown in Figure 2e. This design allows gradients to flow directly through the network, mitigating the potential issues of gradient vanishing or exploding encountered during the training of deep networks.

Residual blocks are typically designed to start with a 3×3 convolutional layer, followed closely by batch normalisation and a ReLU activation function. This is followed by another 3×3 convolutional layer and batch normalisation. To ensure that the dimensionality of the input feature map and the output feature map match, a shortcut path is added to the residual block, which usually contains a 1×1 convolutional layer and batch normalisation, to adjust the dimensionality of the input feature map to sum with the output feature map of the main path. This final summation of the input feature maps with the output feature maps helps to improve the stability and effectiveness of training. The residual function simplifies the optimisation objective and allows the network to focus on learning the residual mapping rather than the entire mapping, which eliminates the need for any additional parameters and thus improves performance. In this way, the residual block is able to propagate the gradient efficiently even when the network is very deep and allows the model to be trained more consistently, ultimately achieving better performance.

### 3.5 Channels and spatial attention mechanisms

Channel attention and spatial attention mechanisms are two key techniques used in deep learning to enhance the feature representation capabilities of models [33]. They improve the convolutional neural network's (CNN) understanding of image content by identifying and enhancing the most important features. Figure 2b shows the structure of the proposed channel–spatial attention block. The channel attention mechanism focuses on assigning different weights on the feature channel dimensions, enabling the network to recognise which channels contain the most useful information for the task at hand. This mechanism assigns a weight to each channel by aggregating statistical information between channels, emphasising informative features and suppressing noisy or irrelevant channels. The spatial attention module, on the other hand, works on the spatial dimension by recognising critical regions in the image and assigning higher weights to these regions. In this way, the model can focus its resources on the most important parts of the image and ignore the less important background or distracting regions. Spatial attention is typically achieved by computing a weighted average of spatial features which generates an attention map that highlights key features in the image. The combined use of these two attention mechanisms provides CNNs with a powerful tool for extracting and emphasising the most relevant features in complex image analysis tasks. For example, in medical image segmentation, this can help the model to more accurately identify lesion regions, even though these regions may be small or partially occluded in the image. In this way, the channel‐attention and spatial‐attention mechanisms not only improve the performance of the model, but also enhance its sensitivity to subtle and critical features, which are essential for accurate image understanding and analysis.

### 3.6 Loss function

In the process of optimising machine learning and deep learning models, the choice of loss function plays a pivotal role. In order to improve the generation of saliency maps with high quality and clarity, we innovatively adopt a hybrid strategy, which skilfully integrates the binary cross‐entropy loss [34] with the Dice loss [35], in order to achieve better training results. Formally, the proposed loss function is defined as follows:

(5) $$L_{\text{total}}=\alpha L_{\text{BCE}}+\beta L_{\text{Dice}}$$

where $L_{\text{BCE}}$ is the binary cross‐entropy loss function, defined as follows:

(6) $$L_{\text{BCE}}=-\frac{1}{N}\sum_{i=1}^{N}\left[y_{i}\;\log\left(p_{i}\right)+\left(1-y_{i}\right)\log\left(1-p_{i}\right)\right]$$

A small value of Equation (6) indicates accurate model predictions. The unique advantage of binary cross entropy (BCE) is reflected in its ability to handle unbalanced datasets, while demonstrating superior stability during optimisation, which is particularly critical for improving model performance.

$L_{\text{BCE}}$ in Equation (5) is the Dice loss function, which focuses on optimising the degree of overlap of the target region when dealing with the category imbalance problem in image segmentation tasks, rather than simply pursuing classification accuracy at the pixel level. $L_{\text{Dice}}$ is defined as follows:

(7) $$L_{\text{Dice}}=1-\frac{2\sum_{i=1}^{N}y_{i}p_{i}}{\sum_{i=1}^{N}y_{i}+\sum_{i=1}^{N}p_{i}}$$

The smaller the Dice loss value, the higher the similarity between the predicted and actual results.

## 4 EXPERIMENTS

### 4.1 Implementation details

We implemented our DDANet using the Pytorch framework and perform experiments on an NVIDIA A100 GPU system. The backbone network Resnet‐34 was pre‐trained on ImageNet. Using the Adam optimiser, the learning rate was set to 1e‐4, the batch size was set to 4, and the maximum number of training cycles was 150. To maintain fairness in comparisons, we used the same hyperparameters to normalise the training of all models. This methodology allows us to systematically refine the models layer by layer, thus ensuring that each decoder layer produces increasingly accurate and unique saliency maps. In implementing the optimal model parameter preservation strategy, we validated the model using a test set after each epoch of training. Specifically, if the Dice loss on the test set exceeds the previously recorded optimal Dice result, we update this optimal value and save the current model parameters. We then load these optimised parameters onto the clinical dataset for further testing. To ensure a fair comparison, we conducted optimal model acquisition for various methods, including U‐Net, Attention U‐Net, and CE‐Net.

**FIGURE 3:**
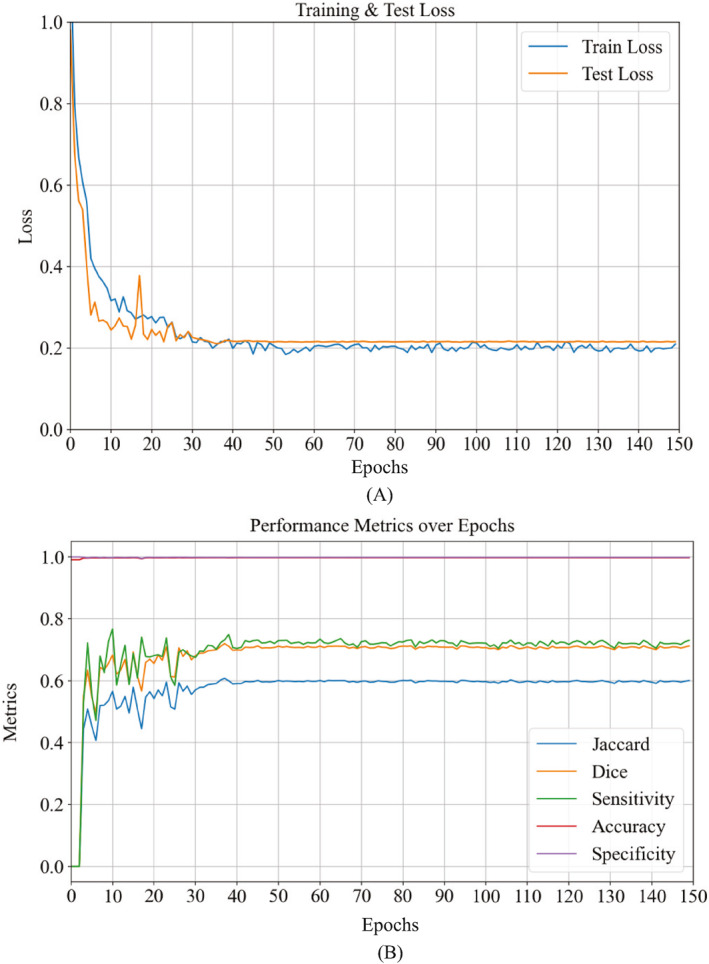
Training and test procedure. (a) The training and test loss for the proposed method and (b) the evaluation metrics used during the testing phase.

As shown in Figure 3, during the training and testing process, the loss value gradually decreased and stabilised with the increase of the number of epochs (Figure 3a), while the evaluation index gradually reached a steady state (Figure 3b), which indicated the good convergence of our DDANet. In addition, we adopt a systematic approach to monitor and optimise the training process of the model. After each epoch, we not only pay attention to the decrease of the loss value but also closely monitor the changes of the evaluation metrics to ensure that the model achieves the best balance in learning and generalisation. With this approach, we can ensure that at the end of model training, we get fully optimised model parameters with the best performance. This strategy not only improves the segmentation precision of the model, but also enhances the reliability and validity of the model in practical applications.

### 4.2 Evaluation metrics

In order to comprehensively evaluate the performance of our DDANet in the ICH segmentation task, several quantitative evaluation metrics were employed including the Dice coefficient (Dice) [36], Jaccard index (Jac) [37], sensitivity (Sen) [38], specificity (Spe) [39], accuracy (Acc) [40]. These evaluation metrics are widely recognised and used in the field of medical image segmentation.

Let *TP* (True Positives) and *FN* (False Negatives) represent the correctly and incorrectly segmented pixel points in the lesion region, respectively, and assume that *TN* (True Negatives) and *FP* (False Positives) represent the correctly and incorrectly segmented pixel points in the non‐lesion region, respectively. The Dice coefficient (Dice) and the Jaccard index (Jac) are the statistical measures used to assess the similarity between the prediction result and ground truth, which are defined as follows:

(8) $$\text{Dice}=\frac{2TP}{2TP+FP+FN}$$

(9) $$\text{Jac}=\frac{TP}{TP+FP+FN}$$

Both Dice and Jaccard range from 0 to 1, the closer to 1.0, the better segmentation performance is. Sensitivity (Sen) is an important evaluation metric that measures the model's ability to correctly recognise ICH pixels, which is defined as follows:

(10) $$\text{Sen}=\frac{TP}{TP+FN}$$

High sensitivity means that the model is able to accurately detect almost all haemorrhagic regions and is critical for clinical diagnosis as it helps to ensure that no important pathologic information is missed. Specificity (Spe), on the other hand, reflects the model's ability to distinguish non‐bleeding regions, defined as follows:

(11) $$\text{Spe}=\frac{TN}{FP+TN}$$

High specificity indicates that the model is able to reduce false‐positive detections, which is equally important to avoid overdiagnosis and unnecessary medical interventions. Accuracy (Acc) provides an overall view of how well the model segment the entire image at the pixel level, which is defined as follows:

(12) $$\text{Acc}=\frac{TP+FN}{TP+FN+FP+FN}$$

The level of accuracy directly affects the reliability of the segmentation results and is an important indicator for evaluating the overall performance of the model.

The combined use of these evaluation metrics can assess the performance of the segmentation algorithm from multiple perspectives, ensuring that our DDANet not only performs well in identifying ICH regions, but also has high accuracy in distinguishing between normal brain tissue and other non‐haemorrhagic regions. Through the quantitative analysis of these metrics, we can more confidently evaluate and compare the advantages and limitations of different algorithms, providing a scientific and objective basis for clinical application.

### 4.3 Comparative experiments

In this section, to objectively demonstrate the effectiveness of our approach, we compare our proposed DDANet with four other methods, that is, U‐Net [11], Attention U‐Net [20], CE‐Net [25], and IHA‐Net [22]. For a fair comparison, we run these methods with default parameters followed Ref. [22].

#### 4.3.1 Quantitative comparison

Table 1 shows the corresponding results of these methods. As shown in Table 1, DDANet achieved the best performance with a Dice coefficient of 0.712, a Jaccard index of 0.601, a sensitivity of 0.730, an accuracy of 0.997, and a specificity of 0.998. Among the networks, IHA‐Net was the closest competitor to DDANet. However, our DDANet surpassed IHA‐Net by 2.45% in the Dice coefficient, 3.26% in the Jaccard index, 2.38% in sensitivity, and performed similar to IHA‐Net in the accuracy and specificity. These results indicated that our DDANet performed excellent precision and edge recognition in the ICH segmentation task.

**TABLE 1:** Performance comparison of different segmentation methods. Bold values indicate that the corresponding methods achieve the highest performance in the corresponding evaluation metrics.

Method	Dice	Jaccard	Sensitivity	Accuracy	Specificity
U‐Net [11]	0.589 ± 0.293	0.475 ± 0.270	0.642 ± 0.321	0.995 ± 0.004	0.997 ± 0.003
Attention U‐Net [20]	0.601 ± 0.279	0.482 ± 0.268	0.577 ± 0.287	0.996 ± 0.004	0.998 ± 0.002
CE‐Net [25]	0.639 ± 0.314	0.532 ± 0.292	0.639 ± 0.339	0.996 ± 0.003	0.998 ± 0.001
IHA‐Net [22]	0.695 ± 0.208	0.582 ± 0.197	0.713 ± 0.231	0.997 ± 0.003	0.998 ± 0.001
Our DDANet	**0.712 ± 0.223**	**0.601 ± 0.240**	**0.730 ± 0.210**	**0.997 ± 0.002**	**0.998 ± 0.002**

#### 4.3.2 Visualisation analysis

Compared to other methods, our proposed DDANet model demonstrates superior performance in several aspects. Firstly, DDANet highlights bleeding areas, which is crucial for medical diagnosis. Secondly, DDANet excels in its ability to identify small target areas, which are often difficult for traditional methods to detect accurately due to their small size.

**FIGURE 4:**
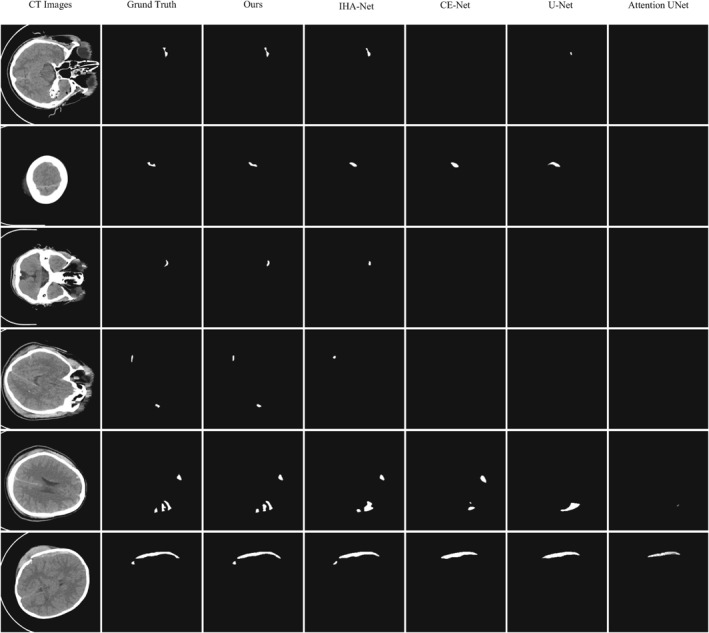
The visual segmentation results comparison chart for tiny lesion cases. From left to right: the original CT images, their corresponding ground truth masks, and the results obtained by our DDANet, IHA‐Net, CE‐Net, U‐Net and Attention UNet, respectively.

As shown in the visualisation results of Figure 4, traditional models like U‐Net, Attention U‐Net, IHA‐Net, and CE‐Net often struggle with small lesion areas, whereas DDANet can clearly identify and segment these regions, significantly improving segmentation precision. Additionally, DDANet also demonstrates strong performance in segmenting irregular regions within images. These regions are typically challenging for traditional algorithms due to their complex and variable shapes. However, through its advanced network architecture and algorithm optimisation, DDANet accurately captures the boundaries of these irregular areas, achieving high‐quality segmentation results. This capability is particularly important in the field of medical image analysis, as it helps to improve the precision and reliability of disease diagnosis, thereby providing better treatment options for patients. Overall, DDANet shows great potential and application value in enhancing segmentation precision and identifying small target areas.

#### 4.3.3 Running efficiency

To comprehensively demonstrate the efficiency of our DDANet, we thoroughly evaluated computational costs including training time, total number of network parameters, and testing time. These metrics reflect the algorithm's operational efficiency and resource consumption in practical applications. Additionally, we recorded the number of epochs required for the model to achieve optimal performance, allowing us to assess the convergence speed and training stability of the model. As shown in Table 2, our DDANet achieved the best detection speed. Furthermore, our model outperformed other methods on all evaluation metrics.

**TABLE 2:** Comparison of parameters for different methods including the number of convergence epoches, training time per epoch, test time per slice, training time, and the total number of parameters.

Method metrics	Convergence epoches	Training time per epoch (s)	Test time per slice (s)	Training time (min)	Total number of parameters (M)
U‐Net [11]	90	44	1.6874	110	13.40 M
Attention U‐Net [20]	95	72	2.4253	180	34.88 M
CE‐Net [25]	75	23	1.0333	57.5	29.00 M
IHA‐Net [22]	40	27	0.9672	67.5	32.00 M
Our DDANet	45	28	0.8763	72	33.00 M

### 4.4 Ablation study

In this section, we conducted ablation experiments including loss ablation and architecture ablation. The loss ablation is to evaluate the roles of BCE and Dice of the proposed loss function defined in Equation (5). The architecture ablation aims to evaluate the key components of our DDANet and the layers of the backbone network ResNet34.

#### 4.4.1 Loss ablation

In this study, we deeply explore the pivotal role of the multi‐objective optimisation function in enhancing the performance of the model. As shown in Table 3, by adjusting the trade‐off parameters α and β, the model can achieved the optimal performance when both α and β were set to 1. This observation is instructive for understanding the importance of balancing between different objectives and also provides an important parameter selection basis for subsequent studies.

**TABLE 3:** The impact of parameters α,β of the multi‐object optimisation function on model performance. Bold values indicate that the corresponding methods achieve the highest performance in the corresponding evaluation metrics.

Multi‐object function	α	β	Dice	Jaccard	Sensitivity	Accuracy	Specificity
$\alpha L_{\text{BCE}}+\beta L_{\text{Dice}}$	0	1.0	0.584 ± 0.231	0.553 ± 0.270	0.632 ± 0.231	0.995 ± 0.004	0.997 ± 0.003
	0.2	0.8	0.601 ± 0.279	0.567 ± 0.268	0.645 ± 0.287	0.996 ± 0.003	0.998 ± 0.002
	0.4	0.6	0.639 ± 0.264	0.532 ± 0.292	0.639 ± 0.210	0.996 ± 0.003	0.998 ± 0.001
	0.6	0.4	0.698 ± 0.238	0.584 ± 0.246	0.693 ± 0.206	0.997 ± 0.002	0.997 ± 0.002
	0.8	0.2	0.695 ± 0.208	0.582 ± 0.246	0.713 ± 0.231	0.997 ± 0.003	0.997 ± 0.001
	0.9	0.1	0.701 ± 0.210	0.579 ± 0.235	0.719 ± 0.201	0.997 ± 0.002	0.998 ± 0.002
	1.0	1.0	**0.712 ± 0.223**	**0.601 ± 0.240**	**0.730 ± 0.210**	**0.997 ± 0.002**	**0.998 ± 0.002**

#### 4.4.2 Architecture ablation

Table 4 demonstrates the specific impact of the extended convolutional pooling (DCP) block on the model performance. The DCP block achieved deeper feature mining by introducing a series of alexial convolutional cascades after different downsampling layers of the encoder in conjunction with the residual module. Especially when the DCP block was combined with the self‐attention mechanism, that is, DCP + Self‐Attention, the performance of the model was further improved. This result not only proved the effectiveness of the DCP block in feature extraction, but also demonstrated the potential of the self‐attention mechanism in improving model performance.

**TABLE 4:** The impact of dilated convolution pooling (DCP) block and self‐attention (SAM) on model performance. Bold values indicate that the corresponding methods achieve the highest performance in the corresponding evaluation metrics.

Multi‐object function	Module	Dice	Jaccard	Sensitivity	Accuracy	Specificity
$\alpha L_{\text{BCE}}+\beta L_{\text{Dice}}$ (α=1.0,β=1.0)	without DCP	0.663 ± 0.231	0.553 ± 0.270	0.663 ± 0.231	0.995 ± 0.004	0.997 ± 0.003
	without SAM	0.654 ± 0.279	0.543 ± 0.268	0.624 ± 0.254	0.996 ± 0.003	0.998 ± 0.002
	without DCP and SAM	0.593 ± 0.210	0.501 ± 0.258	0.629 ± 0.210	0.994 ± 0.002	0.996 ± 0.002
	DCP and SAM	**0.712 ± 0.223**	**0.601 ± 0.240**	**0.730 ± 0.210**	**0.997 ± 0.002**	**0.998 ± 0.002**

We also conducted an in‐depth exploration of the impact of different backbone network layers on model performance. Table 5 shows that as the number of network layers increased, the model's performance improved across several evaluation metrics. These observations further confirmed that appropriately increasing network depth helps extract more comprehensive features, thereby enhancing the overall performance of the model in the task of ICH image segmentation.

**TABLE 5:** Impact of the number of backbone network layers on model performance. Bold values indicate that the corresponding methods achieve the highest performance in the corresponding evaluation metrics.

Backbone	Layers	Dice	Jaccard	Sensitivity	Accuracy	Specificity
Resnet 50	Layer 3	0.654 ± 0.228	0.547 ± 0.269	0.674 ± 0.227	0.994 ± 0.001	0.994 ± 0.002
	Layer 4	0.689 ± 0.274	0.563 ± 0.271	0.689 ± 0.249	0.996 ± 0.002	0.997 ± 0.002
	Layer 5	0.701 ± 0.218	0.594 ± 0.258	0.713 ± 0.219	**0.998 ± 0.002**	0.997 ± 0.002
Resnet 34	Layer 3	0.694 ± 0.217	0.554 ± 0.233	0.698 ± 0.229	0.995 ± 0.001	0.994 ± 0.001
	Layer 4	0.710 ± 0.289	0.594 ± 0.245	0.719 ± 0.214	0.997 ± 0.001	**0.998 ± 0.002**
	Layer 5 (ours)	**0.712 ± 0.223**	**0.601 ± 0.240**	**0.730 ± 0.210**	0.997 ± 0.002	**0.998 ± 0.002**

In summary, these experimental results not only provide us with an effective strategy to optimise the model performance but also provide a valuable reference for future model design in similar tasks. Through careful parameter tuning and module innovation, we are able to continuously push the boundaries of model performance to cope with the increasingly complex task of segmenting brain haemorrhage images.

## 5 DISCUSSION

ICH is a medical emergency that can be life‐threatening, typically caused by ruptured blood vessels in the brain, leading to the accumulation of blood in brain tissue. This condition can arise from various factors such as hypertension, head trauma, or vascular malformations, often resulting in severe compression and damage to brain tissue, which can lead to neurological impairment or even death. Therefore, rapidly and accurately detecting and segmenting ICH regions is crucial for patient prognosis.

The DDANet model proposed in this paper demonstrates superior performance in the task of ICH image segmentation, particularly addressing the limitations of existing methods in detecting small lesion areas. Previous studies have struggled with feature information loss due to deep network structures, making it difficult to effectively capture the characteristics of small lesions [11, 20, 22]. By introducing the DCP block and self‐attention mechanisms, we successfully enhanced the model's ability to extract multiscale features, effectively addressing issues related to pixel imbalance and the challenge of detecting small lesion areas.

Firstly, the introduction of the DCP block overcomes a major issue common in prior research, where small lesion areas are often overlooked after multiple down‐sampling stages. By incorporating dilated convolutions in the middle layers of the model, we expanded the receptive field, enabling the model to capture broader contextual information. This design significantly improves the model's ability to detect small lesions, particularly in complex ICH images, allowing for the precise segmentation of irregularly shaped lesions. Additionally, the DCP block effectively mitigates pixel imbalance, further enhancing the model's sensitivity to less frequent categories. Secondly, the integration of self‐attention mechanisms further strengthens the model's understanding of global semantic information, compensating for the limitations of conventional CNNs in capturing long‐range dependencies. This enables DDANet to not only perform well in local detail detection but also to comprehensively consider global image information, exhibiting greater robustness in multiscale feature fusion.

Compared to traditional models like U‐Net and other convolution‐based segmentation models, DDANet demonstrates superior precision in handling small lesion areas, significantly improving segmentation accuracy in complex ICH regions. Moreover, by combining residual networks and attention mechanisms, DDANet addresses the gradient vanishing problem commonly encountered in deep network training. Residual networks ensure the deep transmission of information within the network, while attention mechanisms help guide the model to focus on key areas, enhancing its ability to capture complex structures. This design improves the model's overall performance, especially when dealing with the diverse and complex morphology of ICHs, achieving higher segmentation accuracy and robustness.

However, we also observed that despite DDANet's outstanding performance in most cases, there are still limitations when dealing with certain extreme cases of lesions. For example, when confronting very small and irregularly shaped lesion areas, the model's accuracy may slightly decrease. This is primarily due to the extremely limited pixel information in these areas, making it challenging for the model to effectively capture subtle and complex changes during feature extraction. Additionally, the model's sensitivity (Sensitivity) is relatively lower, which is particularly crucial in medical imaging as it directly reflects the likelihood of missing true ICH cases. If an ICH case is misdiagnosed, it can severely affect the patient's subsequent treatment, which is more critical than overdiagnosis. Therefore, although DDANet excels in accuracy and specificity, reducing false positives and improving overall diagnostic efficiency, the sensitivity issue still requires further optimisation.

To improve sensitivity, future research could focus on the following areas: First, addressing class imbalance through data augmentation and class re‐weighting strategies, thereby enhancing the detection of small haemorrhage regions. Secondly, incorporating multi‐modal data (such as MRI or other clinical data) could further enhance the model's performance. Additionally, future model designs could specifically optimise sensitivity by introducing specialised loss functions or more advanced attention mechanisms, helping the model better focus on critical haemorrhage regions. These improvements could further reduce the risk of missing ICH cases.

In conclusion, the experimental results of this study not only provide effective strategies for optimising model performance but also offer valuable insights for future model design in similar tasks. Through fine‐tuned parameter adjustments and module innovations, we aim to continuously push the boundaries of model performance to tackle increasingly complex ICH image segmentation tasks. These advancements offer new possibilities for the early detection and treatment of ICH and demonstrate the immense potential and applications of deep learning in medical image processing.

## 6 CONCLUSION

In this paper, a brain haemorrhage region segmentation model based on CNN for brain CT images of patients with cerebral haemorrhage is proposed, and the feature extraction ability and information expression ability of the model are improved by introducing deep dilation pooling block (DCP) and self‐attention mechanism. This model further integrates residual networks, channel attention, and spatial attention mechanisms to aggregate features of different levels, effectively mitigating the severe class imbalance problem and aiding the training process. The experimental results show that the proposed model outperforms the existing methods on the internal test data, especially in the evaluation indexes such as the Dice coefficient and Jaccard index, which have achieved significant improvement, respectively, to 0.712 and 0.601. This suggests that the model is able to effectively segment ICH regions, especially in coping with pixel imbalance and the detection of tiny lesion regions, demonstrating strong robustness and precision. Combining the quantitative analysis and visual comparison results, the method in this paper demonstrates good potential for clinical application in the ICH segmentation task and is expected to provide effective support for automated detection and diagnosis of ICH.

## AUTHOR CONTRIBUTIONS

**Haiyan Liu**: Writing ‐ original draft; Writing ‐ review & editing. **Yu Zeng**: Software. **Hao Li**: Data curation; Formal analysis. **Fuxin Wang**: Writing ‐ original draft. **Jianjun Chang**: Supervision; Writing ‐ review & editing. **Huaping Guo**: Methodology; Writing ‐ review & editing. **Jian Zhang**: Supervision; Writing ‐ review & editing.

## CONFLICT OF INTEREST STATEMENT

The authors declare no potential conflicts of interest.

## Data Availability

Our code, dataset (pre‐processed) and experimental results are available at https://github.com/hpguo1982/DDANet.
